# Effectiveness and challenges of DOT in multidrug-resistant TB: a 12-year retrospective study

**DOI:** 10.5588/pha.25.0057

**Published:** 2026-05-18

**Authors:** S. Jabeen, S. Memon, F. Rashid, N. Ahmed, M.N. Khan, S.M. Qadir, M. Akram, N. Ahmed, A.W. Khan, Y. Fu, F. Zhang

**Affiliations:** 1 Department of Microbiology, Wu Lien Teh Institute, Harbin Medical University, Harbin, China;; 2 Institute of Health, Kunming Medical University, Kunming, China;; 3 Department of Bioinformatics and Biosciences, Capital University of Science and Technology, Islamabad, Pakistan;; 4 National Reference Laboratory for Tuberculosis, National TB Control Program, Islamabad, Pakistan;; 5 Department of Cell Biology and Genetics, Shantou University Medical College, Shantou, China;; 6 Department of Life Sciences, School of Science (SSC), University of Management and Technology, Lahore, Pakistan;; 7 Provincial Reference Lab, Ojha Institute of Chest Diseases, Dow University of Health Sciences, Karachi, Pakistan;; 8 Heilongjiang Key Laboratory of Immunity and Infection, Harbin, China.

**Keywords:** tuberculosis, directly observed therapy, MDR-TB, treatment outcomes, Pakistan, Sindh, long-term regimen, short-term regimen

## Abstract

**SETTING:**

TB remains a major public health problem in Pakistan, a high-burden country with a high prevalence of multidrug-resistant TB (MDR-TB). Directly Observed Treatment (DOT) is widely used to improve treatment adherence; however, provincial-level evaluations remain limited, particularly in Sindh.

**OBJECTIVE:**

To assess the effectiveness, outcomes, and challenges of DOT in Sindh Province over a 12-year period. A retrospective observational study was conducted including all patients enrolled in the National DOT Programme in Sindh from 2010 to 2022. Demographic, clinical, and treatment data were evaluated.

**RESULTS:**

Pulmonary MDR-TB was highly prevalent among individuals aged 16–35 years. Extensively drug-resistant TB (XDR-TB) accounted for 6.32% of cases and pan drug-resistant TB (PDR-TB) for 0.32%. Before enrolling into DOT, most patients (48.35%) had treatment outcomes that were not evaluated or had failed treatment (38.45%) in their previous non-DOT therapy. DOT achieved cure rates of 68% for long-term regimens and 75% for short-term regimens, with timely sputum conversion.

**CONCLUSION:**

DOT was effective for managing MDR-TB; however, high MDR-TB prevalence, delayed diagnosis, treatment-related side effects, and patient loss to follow-up emphasise the need for strengthened early detection, adherence support, and monitoring strategies to improve treatment outcomes.

Even though it is curable and preventable, TB remains a significant global health concern and continues to be the leading cause of mortality due to an infectious agent.^1^ Pakistan has the fifth highest TB burden, and is responsible for 61% of the TB cases in Eastern Mediterranean region,^2^ and fourth in the prevalence of multidrug-resistant TB (MDR-TB).^3^ In 1991, the World Health Assembly declared TB a major global health concern, prompting intensified international action.^4^ The focus was placed on accurate diagnosis and effective treatment. In 1994, the WHO introduced the Directly Observed Treatment Short Course (DOTS) strategy, which ensures supervised medication intake to improve adherence and prevent drug resistance. Current global goals include a 90% reduction in TB deaths by 2030 and 95% by 2050.^5^ Achieving this requires an average annual incidence decline of about 20% between 2015 and 2050, demanding intensified efforts.^5^ Patients receive DOT either at a health facility supervised by a trained provider, or at home under a designated caregiver.

Effective treatment strategy includes proper and accurate diagnosis, followed by antibiotic sensitivity testing, appropriate medication, preventive measures, and follow-up.^6^ However, there is currently no conclusive study published on the successful outcomes of the DOT programme in Pakistan, particularly from Sindh Province. The increased incidence of TB and treatment failures are associated with various factors majorly including misdiagnosis.^7^ Although DOT is considered a cornerstone of TB management, evidence on its long-term impact in Pakistan, especially in Sindh Province, remains limited. Therefore, this study evaluates the effectiveness and challenges of DOT over a 12-year period in Sindh, providing insights into treatment outcomes and contributing factors that can inform future policy and programme improvement. This is the first comprehensive 12-year retrospective analysis of DOT outcomes in Sindh, Pakistan, linking demographic, clinical, and regimen data (long-term treatment regimen vs. short-term treatment regimen).

## METHODS

A retrospective observational study was conducted using data from TB centres working under the National TB DOT Programme of Sindh, Pakistan. Major sites like Sukkur, Larkana, and Jamshoro were included in this study. The study included all patients with MDR-TB diagnosed between 2010 and 2022 and enrolled into the DOT programme, based on the WHO approach to TB control.^8^ Standard definitions were used for terms such as MDR-TB or extensively drug-resistant TB (XDR-TB),^9^ and cure, treatment completed, or loss to follow-up.^10^

### DOT strategy

DOT is a strategy used to make sure that TB patients adhere to their treatment regimen. In this approach, patients are closely monitored either in a clinical setting with a designated person, typically a nurse, ensuring proper medication intake, or at home, where a family member takes responsibility for medication adherence. Involvement of trained health care workers is significant. The current DOT strategy spanned maximum 24 months. Upon enrolment, sputum samples were collected, stored, and tested (GeneXpert and culturing) following WHO protocols.^11^ Treatment continues, with monthly sputum culturing.

### Treatment regimen

Mainly two treatment regimens were followed for the treatment of MDR-TB. One is long-term treatment regimen (LTR) and the second one is short-term treatment regimen (STR). Duration of LTR is from 8 months to 18–24 months and includes levofloxacin (Lfx), bedaquiline (BDQ), linezolid (LNZ), clofazimine (CFZ), cycloserine, pyrazinamide (Z), and amikacin, while STR is only for 9–12 months and includes Lfx/moxifloxacin, CFZ, Z, ethambutol, isoniazid, and ethionamide. The LTR was followed from 2010 till 2019, where some other drugs such as LNZ, CFZ, and BDQ were also included separately or in combination with LTR or excluded depending upon the clinical history and adverse reactions faced during the treatment. However, after 2019 only STR is followed.

### Data collection and processing

Data for all DOT cases from 2010 to 2022 were obtained from the regional DOT office in Sindh, Pakistan. Non-relevant variables were excluded.

### Statistical analysis

Statistical analyses were performed using SPSS v27 and GraphPad Prism v9.0, with χ² and *t* tests used for group comparisons, Kaplan–Meier analysis for time to culture conversion, and significance set at *P* < 0.05.

### Ethical statement

Ethical approval for the current study was obtained from the Directorate General Health Services, Sindh, under No DDGHS(CDC)TB/1510/12 and the Common Management Unit (TB, Malaria and HIV/AIDS) under F.NO.IRB-CMU-2020-10.

## RESULTS

There was no discernible gender difference among the 1,866 patients: 959 (51.4%) were men and 907 (48.6%) were women (Figure Panel A). The majority of patients were between the ages of 16 and 25 (30.97%), followed by those between the ages of 26 and 35 (27%). These age groups had substantially more cases than other age groups (*P* < 0.05) (Figure Panel B). In terms of occupation, 11.5% were students, 18.4% were workers, and 42.3% were stay-at-home parents. The labour group was dominated by men, whereas the stay-at-home group was dominated by women (Supplementary Data Table S1). Across occupations, the majority of cases occurred among individuals aged 16–35 years.

**FIGURE.**
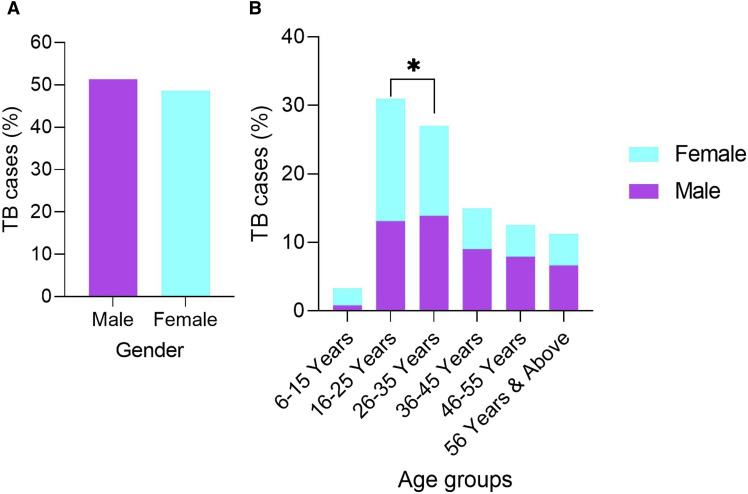
Demographic detail of the patients enrolled in DOT Program. **A)** Detail of the TB cases at gender level. **B)** Detail of the TB cases at different age groups. ^∗^ indicates statistically significant difference between the 16–25 and 26–35 year age groups (*P* < 0.05).

### Details of TB disease

The number of patients enrolled in DOT increased from 57 in 2010 to 239 in 2013, fluctuated in subsequent years, and rose again during 2021–2022 (Supplementary Data Figure S1A). No significant sex-based differences were observed in annual enrolment (Supplementary Data Figure S2A). Across most years, the highest proportion of cases occurred in the 16–25-year group, except in 2013 when the 26–35-year group predominated (Supplementary Data Figure S1A). MDR-TB accounted for 79.1% of cases, followed by mono-drug-resistant TB (13.9%), XDR-TB (6.3%), and pan drug-resistant TB (PDR-TB) (0.6%). Similar patterns were observed across age groups (Supplementary Data Figure S1B) and between men and women (Supplementary Data Figure S2B). Nearly half of the patients (47.6%) were diagnosed within 6 months of symptom onset, and 32.6% after 1–3 years. These patterns were consistent between sexes (Supplementary Data Figure S2C). Across age groups, most diagnoses occurred within 6 months to 3 years, although patients aged 26–35 years more frequently experienced delays exceeding 10 years (Supplementary Data Figure S1C).

### Treatment regimen

A total of 68.3% of patients received LTR, 9.9% received LTR + BDQ + LNZ + CFZ, and 9.6% received STR (Table 1). No significant sex differences were observed in most regimens, except for LTR + LNZ and STR, which were more frequent among men. Across age groups, all regimens were most commonly used in patients aged 16–26 years, while LTR + BDQ was more frequent in those aged 26–35 years. Among pulmonary cases, LTR was the predominant regimen (69.0%), followed by LTR + BDQ + LNZ + CFZ (9.6%) and STR (9.2%) (Supplementary Data Table S2). For extra-pulmonary TB, LTR remained the most common (49.2%), followed by STR (21.5%). By TB type, LTR was used in 46.2% of mono-drug-resistant TB cases and 74.3% of MDR-TB cases, whereas STR was used in 28.9% and 7.0%, respectively. For XDR-TB and PDR-TB, LTR was the main regimen and STR was not used (Supplementary Data Table S3).

**TABLE 1.** Detail of the treatment regimens used in the Directly Observed Treatment programme.

Treatment regimens	Total, n (%)	Gender-wise comparison			Age group–wise comparison						
		Men, n (%)	Women, n (%)	DF, χ²	6–15 years, n (%)	16–25 years, n (%)	26–35 years, n (%)	36–45 years, n (%)	46–55 years, n (%)	56 years and above, n (%)	DF, χ²
LTR	1,275 (68.33)	634 (49.73)	641 (50.27)	3 (0.8446)	45 (3.53)	405 (31.76)	339 (26.59)	193 (15.14)	155 (12.16)	138 (10.82)	**3 (<0.0001)**
LTR + BDQ	63 (3.38)	37 (58.73)	26 (41.27)	3 (0.2074)	1 (1.59)	13 (20.63)	26 (41.27)	8 (12.7)	6 (9.52)	9 (14.29)	**3 (<0.0001)**
LTR + BDQ + LNZ + CFZ	184 (9.86)	88 (47.83)	96 (52.17)	3 (0.6059)	2 (1.09)	65 (35.33)	50 (27.17)	28 (15.22)	25 (13.59)	14 (7.61)	**3 (<0.0001)**
LTR + CFZ	13 (0.7)	5 (38.46)	8 (61.54)	3 (0.5811)	0 (0)	0 (0)	5 (38.46)	1 (7.69)	1 (7.69)	6 (46.15)	**3 (0.0066)**
LTR + LNZ	152 (8.15)	89 (58.55)	63 (41.45)	**3 (0.0352)**	7 (4.61)	53 (34.87)	39 (25.66)	19 (12.5)	13 (8.55)	21 (13.82)	**3 (<0.0001)**
STR	179 (9.59)	106 (59.22)	73 (40.78)	**3 (0.0165)**	7 (3.91)	42 (23.46)	45 (25.14)	30 (16.76)	34 (18.99)	21 (11.73)	**3 (<0.0001)**

Bold text denotes significant results with *P* value < 0.05.

DF = degree of freedom; χ² = chi square; LTR = long-term treatment regimen; BDQ = bedaquiline; LNZ = linezolid; CFZ = clofazimine; STR = short-term treatment regimen.

### Effect of DOT on MDR-TB

The impact of DOT treatment on sputum conversion showed that in LTR, most positive cultures turned negative in the fourth month (17.5%), followed by the sixth month (17.4%) and fifth month (15.4%), while early conversion in the first and second months was lower (6.8% and 6.7%, respectively) – Supplementary Data Figure S3A. In STR, the highest conversion occurred in the third month (35.2%), followed by the fourth month (24.0%) and second month (12.8%) (Supplementary Data Figure S3B). Overall treatment outcomes among 1,869 patients indicated that 55.5% were cured, 13.4% died, 13.2% were lost to follow-up, 9.3% were still under treatment, 3.8% failed, and 3.4% were not evaluated (Supplementary Data Figure S3C). Female patients predominated among those cured or deceased, while men were more represented in lost to follow-up, failed, not evaluated, and still under treatment groups (Supplementary Data Figure S4). The highest number of cured patients were aged 16–25 years, whereas other outcomes were most frequent in the 26–35-year age group (Supplementary Data Figure S3C). Among 179 patients with pre-existing medical conditions, 45.8% were cured, with diabetes, hepatitis C virus (HCV) infection, and hypertension being the most common conditions (Supplementary Data Figure S5A; Supplementary Data Table S4). Patients with behavioural risk factors showed similar trends, with 53.3% cured and 4.4% failing treatment, and smoking was the most frequent behaviour (Supplementary Data Figure S5B; Supplementary Data Table S4). In the 12 patients with both medical and behavioural histories, 6 were cured and 1 failed (Supplementary Data Figure S5C; Supplementary Data Table S4). Side effects were observed in 29.4% of cases, most commonly gastritis (12.1%), joint pain (4.7%), and hearing toxicity (4.4%), while the majority (70.6%) experienced no side effects (Supplementary Data Figure S3D).

### Outcomes of DOT

The outcome of the DOT is evaluated as either successful or unsuccessful. The outcome ‘completed’ and ‘cured’ were counted as successful while ‘failed’, ‘lost follow-up’, ‘not evaluated’, and ‘died’ were taken as unsuccessful. It was observed that the treatment success rate was 68% when LTR was applied, and it was 75% when STR was applied (Supplementary Data Figure S6A). There was no significant difference between both the strategies. In the case of LTR, on average, the sputum culture turned negative at the fifth month while in the case of STR it turned negative at the fourth month. There was no significant difference between both the regimens (Supplementary Data Figure S6B). Detailed outcome of the treatment regimens is presented in Table 2.

**TABLE 2.** Detailed outcome of Directly Observed Treatment regimens.

Outcome	Total, n = 1,866	Male, n (%)	Female, n (%)	6–15 years, n (%)	16–25 years, n (%)	26–35 years, n (%)	36–45 years, n (%)	46–55 years, n (%)	56 years and above, n (%)
Successful									
Cured	1,037 (55.57)	490 (47.25)	547 (52.74)	40 (3.85)	383 (36.93)	288 (27.78)	139 (13.40)	117 (11.28)	70 (6.75)
Complete	26 (1.39)	13 (50.00)	13 (50.00)	5 (19.23)	8 (30.76)	7 (26.92)	4 (15.38)	2 (7.69)	0 (0)
Unsuccessful									
Died	251 (13.45)	112 (44.62)	139 (55.38)	7 (2.79)	54 (21.51)	65 (25.89)	30 (11.95)	35 (13.94)	60 (23.90)
Lost to follow-up	246 (13.18)	171 (69.51)	75 (30.49)	5 (2.03)	54 (21.95)	66 (26.83)	49 (19.92)	33 (13.41)	39 (15.85)
Not evaluated	63 (3.37)	36 (57.14)	27 (42.86)	1 (1.58)	20 (31.75)	14 (22.22)	11 (17.46)	12 (19.04)	5 (7.94)
Failed	70 (3.75)	43 (61.43)	27 (38.57)	1 (1.43)	19 (27.14)	24 (34.28)	10 (14.28)	9 (12.86)	7 (10.00)
Yet to be decided									
Still under treatment	173 (9.27)	94 (54.33)	79 (45.66)	3 (1.73)	40 (23.12)	40 (23.12)	36 (20.80)	26 (15.03)	28 (16.18)

## DISCUSSION

Tuberculosis (TB) remains a major health concern in Pakistan, ranking fifth among high-burden countries and accounting for 61% of TB cases in the Eastern Mediterranean region, with a high prevalence of multidrug-resistant TB (MDR-TB).^12^ DOT is widely implemented to ensure treatment adherence, yet detailed studies are needed to evaluate its effectiveness and identify areas for improvement. This study provides a comprehensive analysis of DOT effectiveness and associated factors in Sindh, Pakistan, from 2010 to 2022. Demographic analysis revealed a balanced gender distribution, consistent with previous reports.^13^ Young adults, particularly those aged 16–25, exhibited higher TB prevalence, corroborating prior findings.^14^ Occupational trends showed higher infection rates among stay-at-home individuals, labourers, and students. Female predominance in the stay-at-home group and male predominance in labourers highlight the role of socio-economic and occupational factors in TB risk.^15,16^ Enrolment trends fluctuated over time, with declines in 2020 likely due to COVID-19 lockdowns, followed by subsequent increases, reflecting changes in awareness, health care access, and policy.^17,18^ Drug resistance analysis indicated a high prevalence of MDR-TB (79.11%), with XDR (6.32%) and PDR (0.59%) also present, underscoring the challenge of treatment and control.^19–22^ Early diagnosis occurred in 47.6% of cases, though delays of up to 10 years were also observed, highlighting the need for timely detection to prevent transmission and drug resistance.^23,24^ Evaluation of DOT regimens revealed that LTR (2010–2019) and STR (post-2019) were both effective. Sputum conversion occurred mostly in the fourth, fifth, and sixth months for LTR and the third and fourth months for STR (Supplementary Data Figures S3A and S3B).^25,26^ Overall, 55.54% of patients were cured, though 13.44% died and 13.18% were lost to follow-up, emphasising adherence and disease severity as ongoing challenges.^27^ DOT was effective across patients with medical and behavioural histories, consistent with previous studies in patients with diabetes or smoking habits.^28,29^ Results for serious comorbidities such as HCV and HIV infection were inconclusive due to small numbers.^30^ Side effects were reported in 29.4% of patients, mainly gastritis (12.1%), joint pain (4.7%), and hearing toxicity (4.4%), aligning with previous findings.^31^ Age-specific analysis showed the highest cure rate in 16–25-year-olds, while older age groups had higher mortality and treatment failure, suggesting an influence of comorbidities and adherence challenges.^32^ DOT success rates were comparable between regimens (LTR 68% and STR 75%) with no significant difference, in agreement with recent findings from Bangladesh.^33^ Key limitations include the single-province setting, incomplete outcome data, limited adjustment for confounders, and insufficient exploration of causes of mortality and loss to follow-up. Overall, this study highlights the effectiveness of DOT in Sindh, with favourable cure rates and sputum conversion. However, high MDR-TB prevalence, delayed diagnosis, treatment-related side effects, and gaps in adherence indicate areas where targeted interventions, policy improvements, and enhanced monitoring are needed.

## Supplementary Material
