# Supplementary material for: Effectiveness and challenges of DOT in multidrug-resistant TB: a 12-year retrospective study
Source: Public Health Action. 2026 May 18;16(2):75–80. doi: 10.5588/pha.25.0057 (PMC13182882; doi:10.5588/pha.25.0057)
Supplement: Supplementary file 1 [file pha25-0057_supplementarydata1.pdf]

Supplementary Material Tables

Table S1: Detail of the occupations of the drug resistant tuberculosis patients studied in current study.

	**Total**	**Gender wise distribution**			**Age groups wise distribution**						
**Occupation**	n (100)	Male n (%)	Female n (%)	DF, X^2	1-15 Years n (%)	16-25 Years n (%)	26-35 Years n (%)	36-45 Years n (%)	46-55 Years n (%)	>56 Years n (%)	DF, X^2
Agriculture	154 (8.25)	145 (94.16)	9 (5.84)	3, (<**0.0001**)	0 (0)	28 (18.18)	52 (33.77)	24 (15.58)	21 (13.64)	29 (18.83)	3, (<**0.0001**)
Beauty Salon	6 (0.32)	6 (100)	0 (0)	3, (**0.0313**)	0 (0)	2 (33.33)	3 (50)	1 (16.67)	0 (0)	0 (0)	3, (0.1562)
Blacksmith	3 (0.16)	3 (100)	0 (0)	3, (0.25)	0 (0)	0 (0)	0 (0)	2 (66.67)	0 (0)	1 (33.33)	3, (0.2206)
Business	99 (5.30)	98 (98.99)	1 (1.01)	3, (<**0.0001**)	0 (0)	24 (24.24)	29 (29.29)	32 (32.32)	8 (8.08)	6 (6.06)	3, (<**0.0001**)
Carpenter	5 (0.27)	5 (100)	0 (0)	3, (0.0625)	0 (0)	0 (0)	0 (0)	2 (40.00)	1 (20.00)	2 (40)	3, (0.3262)
Cattle farm	7 (0.37)	7 (100)	0 (0)	3, (**0.0156**)	0 (0)	2 (28.57)	0 (0)	5 (71.43)	0 (0)	0 (0)	3, (**0.0031**)
Disable	3 (0.16)	3 (100)	0 (0)	3, (0.25)	0 (0)	2 (66.67)	1 (33.33)	0 (0)	0 (0)	0 (0)	3, (0.2206)
Education	17 (0.91)	12 (70.59)	5 (29.41)	3, (0.1435)	0 (0)	4 (23.53)	4 (23.53)	3 (17.65)	5 (29.41)	1 (5.88)	3, (0.2482)
Health worker	18 (0.96)	11 (61.11)	7 (38.89)	3, (0.4807)	0 (0)	3 (16.67)	9 (50)	2 (11.11)	3 (16.67)	1 (5.56)	3, (**0.0052**)
Hotel & Restaurant	9 (0.48)	9 (100)	0 (0)	3, (**0.0039**)	0 (0)	3 (33.33)	3 (33.33)	1 (11.11)	2 (22.22)	0 (0)	3, (0.2751)
Labor 1	245 (13.12)	242 (98.78)	3 (1.22)	3, (<**0.0001**)	1 (0.41)	61 (24.9)	84 (34.29)	40 (16.33)	41 (16.73)	18 (7.35)	3, (<**0.0001**)
Labor 2	98 (5.25)	82 (83.67)	16 (16.33)	3, (<**0.0001**)	1 (1.02)	26 (26.53)	31 (31.63)	21 (21.43)	15 (15.31)	4 (4.08)	3, (<**0.0001**)
Land lord	1 (0.05)	1 (100)	0 (100)	3, (>0.9999)	0 (100)	0 (100)	0 (100)	0 (100)	1 (100)	0 (100)	3, (0.4159)
Preacher	4 (0.21)	3 (75)	1 (25.00)	3, (0.625)	0 (0)	0 (0)	1 (25.00)	1 (25.00)	2 (50.00)	0 (0)	3, (0.4159)
Retired	15 (0.80)	14 (93.33)	1 (6.67)	3, (**0.001**)	0 (0)	0 (0)	1 (6.67)	1 (6.67)	7 (46.67)	6 (40)	3, (**0.0014**)

Security	21 (1.12)	21 (100)	0 (0)	3, (<**0.0001**)	0 (0)	1 (4.76)	6 (28.57)	4 (19.05)	8 (38.1)	2 (9.52)	3, (**0.0186**)
Stay at home	789 (42.26)	46 (5.83)	743 (94.17)	3, (<**0.0001**)	20 (2.53)	243 (30.8)	219 (27.76)	107 (13.56)	85 (10.77)	115 (14.58)	3, (<**0.0001)**
Student	215 (11.52)	101 (46.98)	114 (53.02)	3, (0.3764)	39 (18.14)	155 (72.09)	21 (9.77)	0 (0)	0 (0)	0 (0)	3, (<**0.0001**)
Transport	64 (3.43)	62 (96.88)	2 (3.13)	3, (<**0.0001**)	1 (1.56)	6 (9.38)	23 (35.94)	14 (21.88)	16 (25.00)	4 (6.25)	3, (<**0.0001**)
Unemployed	93 (4.98)	88 (94.62)	5 (5.38)	3, (<**0.0001**)	1 (1.08)	15 (16.13)	22 (23.66)	18 (19.35)	19 (20.43)	18 (19.35)	3, (**0.0031**)

DF = degree of freedom, X^2 = Chi Square, Bold text denotes significant results with p value < 0.05.

Table S2: Detail of the DOT treatment regimens used for Pulmonary and extra-pulmonary TB infections

Treatment regime	Pulmonary n (%)	Extra Pulmonary n (%)	Both n (%)
LTR	1235 (69.03)	32 (49.23)	8 (66.67)
LTR+BDQ	62 (3.47)	1 (1.54)	0 (0)
LTR+BDQ+LNZ+CFZ	172 (9.61)	9 (13.85)	3 (25)
LTR+CFZ	13 (0.73)	2 (3.07)	0 (0)
LTR+LNZ	145 (8.11)	7 (10.77)	0 (0)
STR	165 (9.22)	14 (21.54)	0 (0)

LTR: Long Treatment Regimen, BDQ: Bedaquiline, LNZ: Linezolid, CFZ: Clofazimine, STR: Short Treatment Regimen

Table S3: Detail of the DOT treatment regimens used for types of drug resistant TB

Treatment regime	Mono-DRTB n (%)	MDR n (%)	XDR n (%)	PDR n (%)
LTR	120 (46.15)	1097 (74.27)	48 (40.68)	10 (90.91)
LTR+BDQ	9 (3.46)	30 (2.03)	24 (20.34)	0 (0)
LTR+BDQ+LNZ+CFZ	40 (15.38)	138 (9.34)	6 (5.08)	0 (0)
LTR+CFZ	2 (0.77)	4 (0.27)	7 (5.93)	0 (0)
LTR+LNZ	14 (5.38)	104 (7.04)	33 (27.97)	1 (9.09)
STR	75 (28.85)	104 (7.04)	0 (0)	0 (0)

LTR: Long Treatment Regimen, BDQ: Bedaquiline, LNZ: Linezolid, CFZ: Clofazimine, STR: Short Treatment Regimen

Table S4: Impact of DOT treatment regimens on patients with medical and behavioral history

Conditions	Total (n = 179) 100%	Cured n (%)	Complete n (%)	Died n (%)	Lost to follow up n (%)	Not evaluated n (%)	Failed n (%)	Still under treatment n (%)
**Medical condition**								
Anemia	1 (0.56)	0 (0)	0 (0)	1 (100)	0 (0)	0 (0)	0 (0)	0 (0)
Asthma	1 (0.56)	1 (100)	0 (0)	0 (0)	0 (0)	0 (0)	0 (3.08)	0 (0)
Diabetes	130 (72.63)	68 (52.31)	1 (0.77)	17 (13.08)	16 (12.31)	8 (6.15)	4 (0)	16 (12.31)
Diabetes + HCV	3 (1.68)	0 (0)	0 (0)	1 (33.33)	0 (0)	0 (0)	0 (0)	2 (66.67)
Diabetes + Hypertension	6 (3.35)	1 (16.67)	0 (0)	3 (50)	0 (0)	0 (0)	0 (0)	2 (33.33)
Epilepsy	2 (1.12)	0 (0)	0 (0)	0 (0)	0 (0)	0 (0)	0 (0)	2 (100)
Heart disease	2 (1.12)	0 (0)	0 (0)	0 (0)	1 (50)	0 (0)	0 (0)	1 (50)
Hepatitis C	13 (7.26)	5 (38.46)	2 (15.38)	1 (7.69)	0 (0)	1 (7.69)	0 (0)	4 (30.77)
HIV	3 (1.68)	0 (0)	0 (0)	0 (0)	0 (0)	0 (0)	0 (0)	3 (100)
Hypertension	7 (3.91)	4 (57.14)	0 (0)	3 (42.86)	0 (0)	0 (0)	0 (0)	(0)
Hypothyroid	1 (0.56)	0 (0)	0 (0)	0 (0)	0 (0)	0 (0)	0 (0)	1 (100)
Paralysis	2 (1.12)	0 (0)	0 (0)	0 (0)	0 (0)	0 (0)	0 (0)	2 (100)
Polio	2 (1.12)	0 (0)	0 (0)	0 (0)	0 (0)	1 (0)	0 (33.33)	1 (100)
Psychiatric	3 (1.68)	2 (66.67)	0 (0)	0 (0)	0 (0)	0 (0)	1 (33.33)	0 (0)
Renal disease	3 (1.68)	1 (33.33)	0 (0)	0 (0)	0 (0)	0 (0)	1 (33.33)	0 (0)
**Behavioral Condition**								
Drinker	6 (13.33)	4 (66.67)	0 (0)	0 (0)	1 (16.67)	1 (16.67)	0 (0)	0 (0)
Drug addict	1 (2.22)	0 (0)	0 (0)	0 (0)	1 (100)	0 (0)	0 (0)	0 (0)
Gutka addict	4 (8.89)	1 (25)	0 (0)	0 (0)	2 (50)	0 (25)	1 (25)	0 (0)
Marijuana addict	1 (2.22)	0 (0)	0 (0)	0 (0)	0 (0)	0 (0)	0 (0)	1 (100)
Smoker	27 (60)	15 (55.56)	0 (0)	1 (3.7)	3 (11.11)	3 (11.11)	1 (3.7)	4 (14.81)
Tobacco user	6 (13.33)	4 (66.67)	0 (0)	0 (0)	1 (16.67)	1 (0)	0 (0)	2 (33.33)
**Medical and Behavioral Condition combined**								
Diabetes + Gutka Addict	1 (8.33)	1 (100)	0 (0)	0 (0)	0 (0)	0 (0)	0 (0)	0 (0)
Diabetes + Smoker	7 (58.33)	4 (57.14)	0 (0)	0 (0)	1 (14.29)	1 (14.29)	1 (14.29)	0 (0)
Diabetes + Tobacco	2 (16.67)	1 (50)	0 (0)	0 (0)	0 (0)	0 (0)	1 (50)	0 (0)
Epilepsy + Smoker	2 (16.67)	0 (0)	0 (0)	0 (0)	0 (0)	0 (0)	0 (0)	2 (100)

Supplementary Material Figures

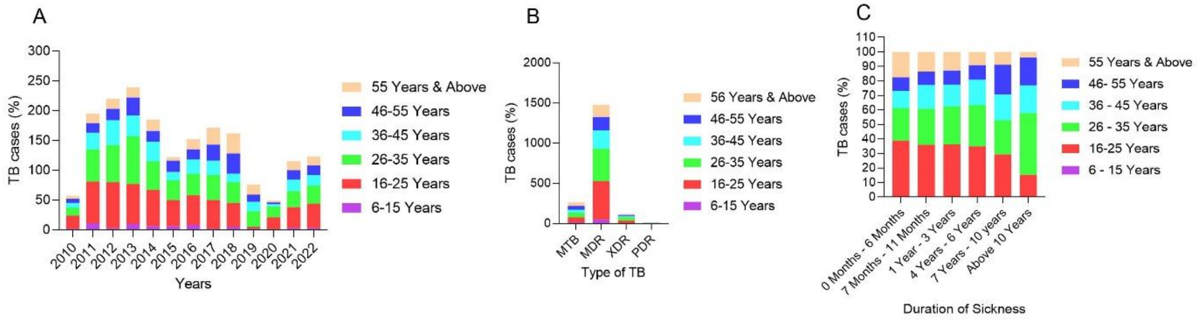

Figure S1: Characteristics of TB patients enrolled in DOT Program (A) Yearly stratification of TB patients enrolled in DOT with different age groups (B) Disease Type stratification in different age groups (C) Type of TB detected in the patients

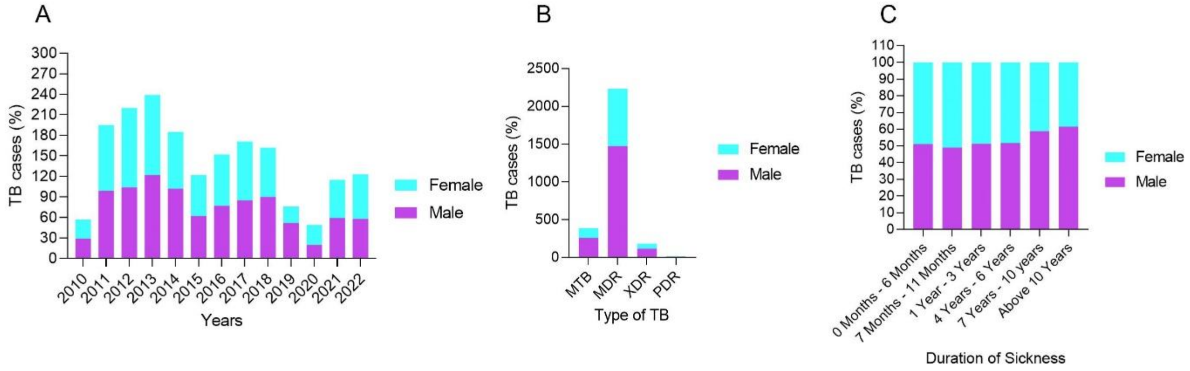

Figure S2: Detailed of the TB disease (A) Detail of the cases in different age groups (B) Number of patients enrolled in to DOT in each year, MTB : Mono drug resistance TB, MDR: Multidrug resistance TB, XDR: Extensively drug resistance TB, PDR : Polydrug resistance TB(C) Stratification of duration of the TB disease in different age groups

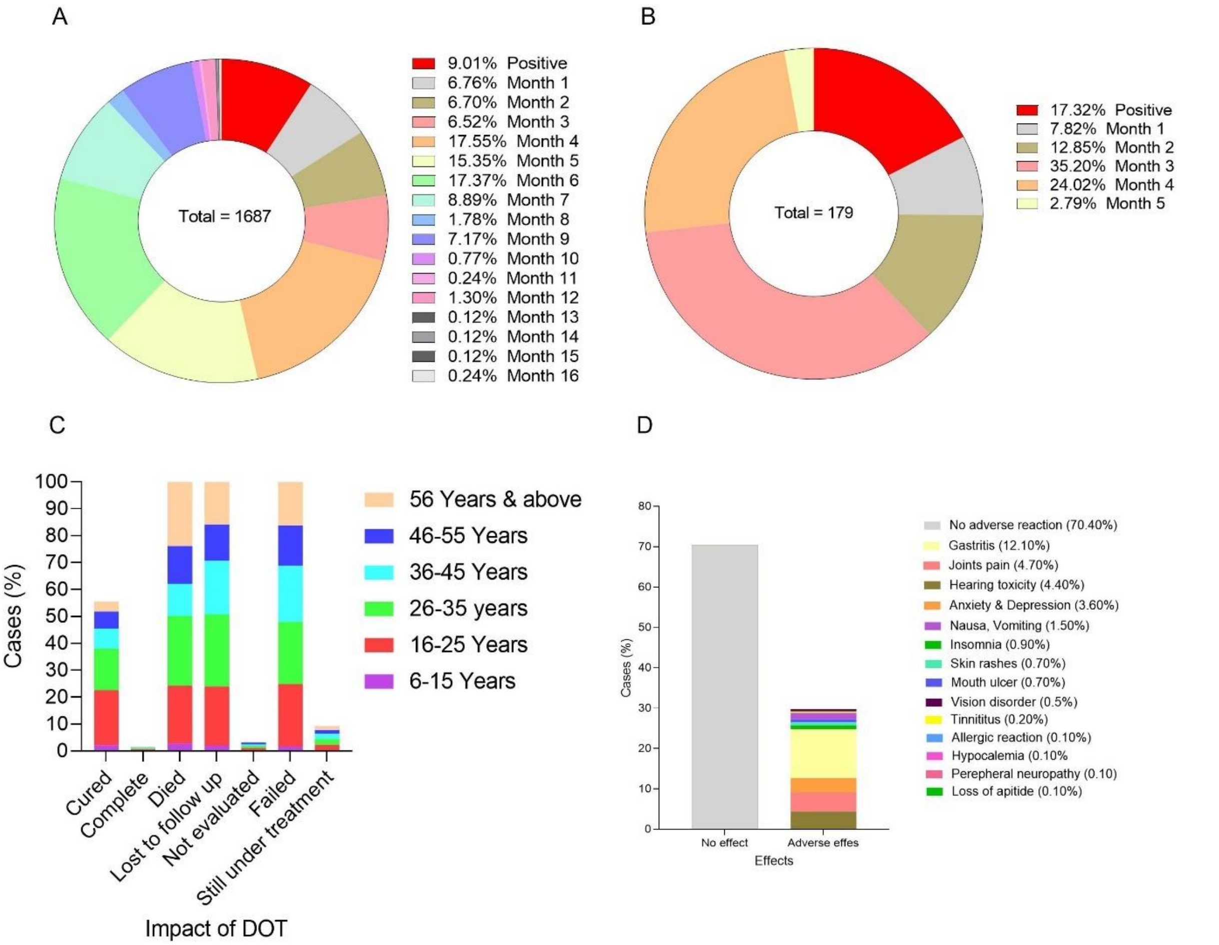

Figure S3: Impact of DOT on TB treatment. (A) Detail of the sputum samples conversion in various months after enrollment into the DOT following the LTR regime (B) Detail of the sputum samples conversion in various months after enrollment into the DOT following the STR regime (C) Outcome of the DOT program (D) Adverse side effects of the DOT treatment.

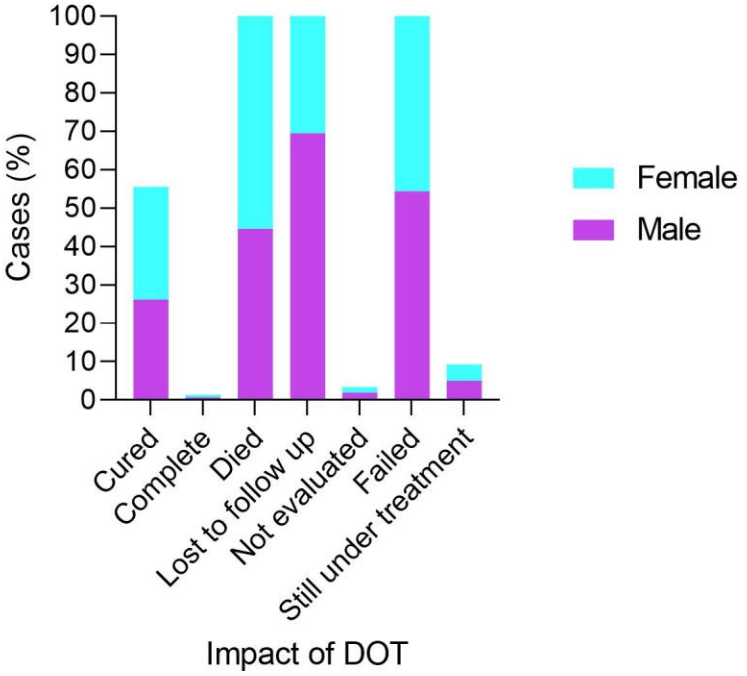

Figure S4: Outcome of DOT treatment at gender level

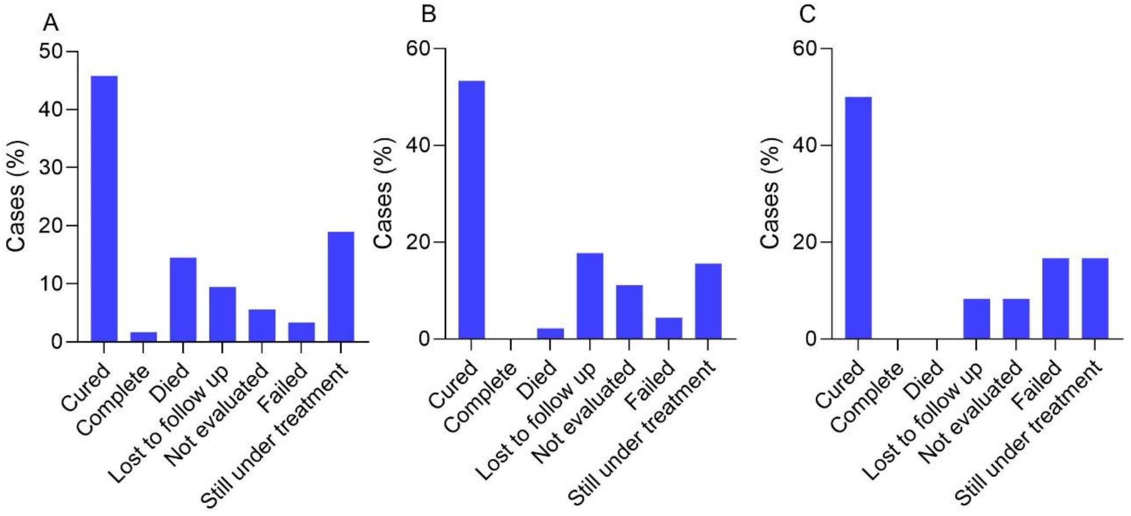

Figure S5: Impact of DOT treatment on patients with medical and behavioral history. (A) Impact of DOT treatment on patients with medical (B) Impact of DOT treatment on patients with

behavioral history (C) Impact of DOT treatment on patients with combined medical and behavioral history

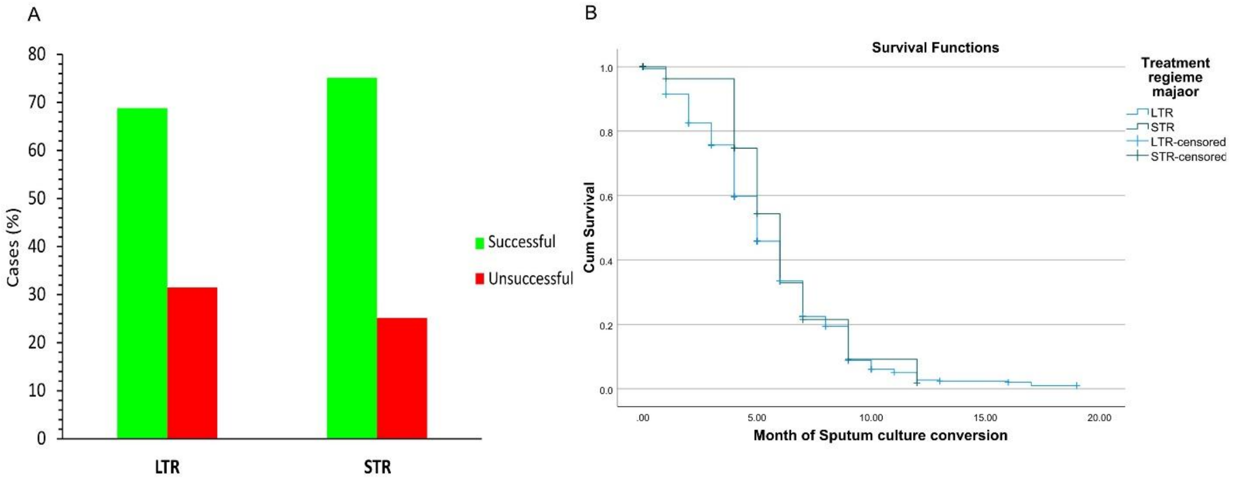

Figure S6: DOT outcome as successful or unsuccessful (A) Percentage of the case whose outcome was successful or unsuccessful completing LTR or STR (B) Time to sputum culture conversion during LTR and STR regimens.
